# Effects of cottonseed hull on intake, digestibility, nitrogen balance, blood metabolites and ingestive behaviour of rams

**DOI:** 10.1038/s41598-023-29005-0

**Published:** 2023-02-08

**Authors:** Anderson M. Zanine, Wanderson J. R. Castro, Daniele J. Ferreira, Alexandre L. Souza, Marinaldo D. Ribeiro, Henrique N. Parente, Michelle O. M. Parente, Edson M. Santos, Juliana S. Oliveira, Anny Graycy V. O. Lima, Thiago V. C. Nascimento, Francisco Naysson S. Santos, Fagton M. Negrão, Cledson G. Sá

**Affiliations:** 1 Department of Animal Science, Federal University of Maranhão, Chapadinha, MA 65500-000 Brazil; 2 Department of Animal Science, Federal University of Mato Grosso, Cuiabá, MT 78735-901 Brazil; 3 Department of Animal Science, Federal University of Goiás, Goiânia, GO 74690-900 Brazil; 4 Department of Animal Science, Federal University of Paraíba, Areia, PB 58397-000 Brazil; 5 Federal Institute of Education, Science and Technology of Rondônia, Colorado do Oeste, RO 76993-000 Brazil

**Keywords:** Zoology, Animal behaviour

## Abstract

The aim of this study was to determine the effect of cottonseed hull (CH) in the diets of rams on intake, digestibility, nitrogen balance, ingestive behaviour, and blood metabolites. Twenty males, uncastrated with an average body weight of 29.08 ± 4.18 kg, were distributed in a completely randomized design with four treatment diets and five replicates. The diet contained a forage to concentrate ratio of 50 : 50 (ground corn, soybean meal, corn silage, and CH), and the experimental treatments were inclusion of 0, 10, 20, and 30% CH (dry matter basis). Inclusion of CH linearly increased water intake, effective intake of ethereal extract and non-fibrous carbohydrates, and crude protein digestibility. Dry matter (DM) and neutral detergent fiber (NDF) digestibility, nitrogen faecal losses, and nitrogen balance reduced linearly with CH inclusion in the diet. Inclusion of CH in rams’ diet reduces intake, nutrient digestibility, and intake and rumination efficiencies. Thus, it is not recommended to add this by-product to the diet of high-performance rams, but for nurture that does not aim at high performance, inclusion of up to 5% of this by-product is recommended.

## Introduction

The high cost of animal feed in confinement systems is undoubtedly one of the biggest barriers to the adoption of this system by farmers. However, alternative feeds included in this scope make the profits of these systems more evident, making them more attractive to those who explore them^1^. However, the viability of using agroindustry by-products in ruminant feed requires research to characterize them, and to determine their nutritional value and the need for purification, as well as conservation, storage, and marketing^2,3^.

Among the several by-products that Brazilian agroindustry offers, cotton is the most used in animal feed because it is a culture that generates a series of by-products such as lump, cake, and meal, as well as linter, cottonseed hull, and delinted cottonseed, which are still little worked and studied in the scientific community.

Cottonseed hull (CH) is obtained during processing for oil production. It is composed of an outer layer of cottonseed with some linter adhered. It has a high content of fibre and lignin (25, 96, 79 and 11%, respectively) unattractive for non-ruminants’ diets, but it may be an interesting option in ruminant feeding^4^.

According to Ref.^5^, cotton lump and CH are by-products rich in neutral detergent fibre that is not derived from forage; that is, due to the processes that they suffer in agroindustry to obtain the primary products, the fibre presents smaller granulometry and so greater gravity^6^, which theoretically result in a shorter feed retention time in the rumen. Thus, research on feeding behaviour becomes a very important tool in diet evaluation through quantification of the time spent feeding, ruminating, and idle^7,8^. In view of the above, we hypothesized that CH can replace traditional sources of forage such as silage or hay, which becomes an interesting alternative from an economic and environmental point of view.

Thus, studies that make inferences about alternative products are of paramount importance to better target strategies within intensive production systems. In this study, the aim was to evaluate the intake, digestibility, nitrogen balance, ingestive behaviour, and blood metabolites of rams fed with CH in the diet.

## Results

Nutrients intake in g/animal/day for all nutritional fractions, decreased linearly (P < 0.05), except for intake of water (L/day) which showed no effect of CH inclusion in the rams’ diet (Table 1).

**Table 1:** Feed intake and apparent digestibility coefficient of rams fed different levels of cottonseed hull.

Item	Cottonseed hull (% DM)				SEM	*P*-value	
	0	10	20	30		L	Q
Daily feed intake (g/day)							
Dry matter^1^	883.86	640.69	555.92	341.86	66.70	0.001	0.097
Organic matter^2^	844.66	612.69	529.97	322.93	66.58	0.001	0.097
Crude protein^3^	101.95	75.03	62.72	58.13	7.17	0.001	0.006
Neutral detergent fiber^4^	363.39	213.58	154.16	66.75	28.44	0.001	0.006
Ethereal extract^5^	24.55	19.29	17.91	12.89	1.75	0.004	0.198
Non-fibrous carbohydrates^6^	453.56	408.99	392.99	331.12	24.44	0.002	0.496
Total carbohydrates^7^	718.16	518.37	449.34	251.91	34.96	0.004	0.052
Water	2.22	2.39	2.07	2.67	284.7	0.43	0.639
Composition of the diet effectively consumed (%)							
Crude protein	11.53	11.71	11.28	17.00	1.70	0.086	0.181
Ethereal extract^8^	2.78	3.01	3.22	3.77	0.14	0.004	0.193
NDF_ap_^9^*	41.11	33.34	27.73	19.53	2.27	< 0.001	0.767
Non-fibrous carbohydrates^10^	51.32	63.84	70.69	96.86	6.86	0.001	0.235
Digestibility coefficient (%)							
Dry matter^11^	70.2	52	55.4	42.2	5.11	0.002	0.159
Crude protein^12^	50.3	63.3	57.3	64.7	2.93	0.011	0.104
Neutral detergent fiber^13^	60.6	40.0	39.9	37.5	2.02	0.001	0.004
Ethereal extract^14^	78.3	66.0	64.9	58.3	3.18	0.004	0.054
Non-fibrous carbohydrates	87.7	85.9	87.2	90.3	1.92	0.309	0.364
Total carbohydrates	43.3	41.2	41.0	39.5	2.60	0.328	0.695

*Neutral detergent fiber corrected for ash and protein.

^1^Y = 862.202 − 17.107X; ^2^Y = 824.759 − 16.479X; ^3^Y = 96.029 − 1.437X; ^4^Y = 341.875 − 9.493X; ^5^Y = 24.120 − 0.363X; ^6^Y = 454.174 − 3.833X; ^7^Y = 291.299 − 6.973X; ^8^Y = 2.6734 + 0.0399X; ^9^Y = 41.334 − 0.7785X; ^10^Y = 47.691 + 1.8113X; ^11^Y = 67.058 − 0.808X; 1^2^Y = 53.306 + 0.373X; ^13^Y = 54.885 − 0.693X; ^14^Y = 76.014 − 0.609X.

Regarding the composition of the diet effectively consumed by animals, it can be observed that there was no significant effect (P > 0.05) on CP from CH inclusion in the animals’ diet. Cottonseed hull inclusion increased linearly the effective intake of EE and NFC (P < 0.05) while the effective intake of NDFap decreased linearly.

Cottonseed hull inclusion in rams’ feed linearly reduced (P < 0.05) the apparent digestibility of DM by 0.80 percentage units for each 1% CH included. The control treatment had 70.2% DM digestibility, while the highest inclusion level of CH had 42.2%. The same behaviour (P < 0.05) was observed for the apparent digestibility of NDF and EE.

The apparent digestibility of CP increased linearly (P = 0.011) as the amount of CH increased in the diet. No significant effect (P > 0.05) was observed for the apparent digestibility of NFC and TC.

Nitrogen balance (NB) decrease linearly (P = 0.001) with CH inclusion in the rams’ diet, reducing by 0.51 g/day for each 1% of CH included (Table 2).

**Table 2:** Nitrogen balance and blood metabolites of rams fed with cottonseed hull levels.

Variables	Cottonseed hull (%DM)				SEM	*P*-value	
	0	10	20	30		L	Q
Nitrogen balance^1^	10.01	6.41	4.5	1.11	2.04	0.001	0.694
Faecal nitrogen^2^	2.71	3.08	3.56	3.51	0.15	0.009	0.027
Urine nitrogen	3.59	2.51	1.98	4.68	0.99	0.546	0.119
Blood urea (mg/dL)	21.3	20.65	17.61	17.39	3.24	0.324	0.729
Glucose (mg/dL)	80.8	65.4	71.8	71.4	6.07	0.433	0.176

^1^Y = 10.13–0.51X; ^2^Y = 2.78 + 0.04X.

Faecal nitrogen (FN) increased linearly (*P* = 0.009) from 0.04 g/day for every 1% of CH included in the animals’ diet, whereas nitrogen lost by urine (UN), plasma urea (BU), and glucose showed no significant effect (P > 0.05) of CH inclusion in rams’ feed, averaging 3.19 g/animal/day, and 19.24 and 72.35 mg/dL, respectively.

The inclusion of CH in the diets affected most variables for the ingestive behaviour measured in time (h) and distributed in the daytime and night-time periods (Table 3).

**Table 3:** Ingestive behaviour data of rams fed different levels of cottonseed hull.

Variables	Cottonseed hull (%DM)				SEM	*P*-value	
	0	10	20	30		L	P
Time spent eating (min/day)	206	169	167	149	0.47	0.444	0.311
Daytime (min/day)	116^a^	109^a^	112^a^	104^a^	0.15	0.477	0.838
Night-time (min/day)^1^	90^b^	60^b^	55^b^	45^b^	0.18	0.011	0.101
Time spent rumination (min/day)^2^	434	437	375	357	0.23	0.005	0.512
Daytime (min/day)^3^	197^a^	177^a^	146^a^	123^a^	0.22	0.001	0.557
Night-time (min/day)	237^b^	260^b^	229^b^	234^b^	0.27	0.579	0.738
Time spent idleness^a^ (min/day)^4^	800	834	898	934	1.06	0.004	0.714
Daytime (min/day)^5^	406^a^	421^a^	463^a^	478^a^	0.32	0.011	0.466
Night-time (min/day)^6^	394^a^	413^a^	435^a^	456^a^	0.28	0.017	0.528
Total chewing time^b^ (min/day)^7^	640	606	542	506	28.8	0.002	0.426
Number of ruminated boluses/day^8^	602	569	516	462	35.3	0.008	0.572
Eating rate g DM/h^9^	257	227	200	138	28.5	0.004	0.695
Eating rate g NDF/h^10^	106	76	55	27	12.1	0.001	0.147
Rumination rate g DM/h^11^	122	88	89	57	10.2	0.006	0.214
Rumination rate g NDF/h^12^	50	29	25	11	4.14	0.001	0.014

^a^Idling time includes all other activities that are not eating or rumination.

^b^Total chewing time includes eating and rumination activities. Means followed by the same letter within the same column and variable are not significantly different (P > 0.05) by t-test.

^1^Y = 1.381 − 0.023X; ^2^Y = 4.493 − 0.034X; ^3^Y = 3.278 − 0.042X; ^4^Y = 9.052 + 0.157X; ^5^Y = 6.622 + 0.041X; ^6^Y = 6.474 + 0.033X; ^7^Y = 635.488 − 4.65X; ^8^Y = 608.625 − 4.742X; ^9^Y = 277.112 − 4.268X; ^10^Y = 110.372 − 2.782X; ^11^Y = 120.341 − 1.955X; ^12^Y = 48.212 − 1.254X.

The inclusion of CH did not affect significantly (P > 0.05) the time spent eating (all day) and in daytime nor time spent rumination in night-time, with mean values of 173, 110 and 240 min/day, respectively.

The animals’ night-time eating, and daytime rumination reduced linearly (P < 0.05), while time spent idleness during daytime and night-time increased (P < 0.05) when CH was included in rams’ feed.

In relation to the period of day, it can be observed that the times spent in daytime feeding and rumination were longer than the night-time ones (P < 0.05), while the time spent idling did not differ statistically (P > 0.05).

All data regarding eating and rumination rate (g DM and NDF/h) decreased linearly (P < 0.05) by inclusion of CH in the rams’ diet.

Time spent idling and the number of ruminated boluses per day increased linearly (P < 0.05), while total chewing time, and time spent ruminating decreased linearly with CH inclusion.

## Discussion

The decrease of nutritional fraction intake may be related to animals’ selectivity, because due to the physical form of CH with the presence of a plume, it does not mix homogeneously with the other ingredients of the diet, resulting, almost exclusively, in intake of concentrate and corn silage.

In studies with cotton by-products^9^ checking 0, 10, 20 and 30% CH in the diet of feedlot cattle, verified that during the initial period of confinement, the animals selectively restricted cottonseed hull; however, during the experiment, this selectivity was reduced, and it did not affect the intake of DM, MO, CP, NDF and NFC (kg/day).

Several authors have also reported an increase in nutrient intake by cattle with diets containing CH^10–12^. In the present study, throughout the period of confinement, rams were more selective than cattle, since they maintained a greater restriction of CH. This behaviour shows the difference in feeding habits of these animals. According to Refs.^13,14^, sheep are highly selective for feed because they have greater lip mobility, while cattle have low selectivity.

The decrease in CP intake can be explained by the decrease in DM intake from animals fed with CH in the diet, because according to Ref.^15^ the amount of dietary nitrogen limits fibrous fraction digestion (Table 2) because it does not allow proper development of ruminal microorganisms, reducing the passage rate to the abomasum and consequently limiting the animal’s intake by filling.

Through the composition of the diet effectively consumed (Table 4), it is possible to prove the animals’ selectivity to the diet offered, because according to the chemical composition of the diets, the EE content was from 4.03 to 3.46%, NFC from 53.08 to 47.19%, and NDFap from 28.45 to 35.47% between 0 and 30% inclusion; when the composition of the diet effectively consumed was observed, the EE content was from 2.78 to 3.77%, NFC from 51.32 to 96.86% and NDF_ap_ from 41.11 to 19.53%. This difference between the diet offered and the composition of the diet effectively consumed indicates that the animals had a predilection for the concentrate and corn silage, because they presented a higher percentage of EE and NFC and a lower one of NDFap, in relation to CH.

**Table 4:** Chemical composition of main ingredients in the experimental diets.

Item	Corn silage	Ingredients (g/kg DM)		Cottonseed hull
		Ground corn	Soybean meal	
Dry matter	328.34	92.01	899.34	900.4
Organic matter	941.23	989.22	933.56	954.4
Ash	58.77	10.78	66.44	45.6
Crude protein	69.98	90.33	493.66	62.34
Ether extract	31.9	54.77	14.34	12.5
NDFap^1^	455.69	113.87	110.54	689.52
ADF^2^	326.33	32.08	84.34	548.6
NFC^3^	383.66	730.29	315.02	190.04
TC^4^	839.35	844.16	425.56	879.56
TDNe^5^	634.78	864.00	823.29	461.64

^1^Neutral detergent fibre corrected for ash and protein.

^2^Acid detergent fibre.

^3^Non-fibrous carbohydrate.

^4^Total carbohydrate.

^5^Total digestive nutrient estimate.

The decrease in EE and NFC intake, may be related to the decrease in DM intake associated with a decrease of these nutrients on inclusion of CH in diets (Table 6). According to Ref.^13^, diets containing more than 7% EE can affect DM intake by energy density, an observation that cannot be attributed to this research because the experimental diets showed maximum concentrations of 4% EE.

Even with the decrease in CP and EE intake, the nutritional fractions used for the calculation of TC, the ash intake increased with the inclusion of CH in the animals’ diets, which prevented an increase in TC intake.

The diets did not significantly affect the animals’ water intake, which showed a mean of 2.34 L. This result is lower than that recommended by Ref.^8^ of 2.870 L/animal, which can be associated with a low DM intake because it has a direct influence on this variable.

The decrease in DM digestibility on inclusion of CH in rams’ diets may be associated with the high C fraction of carbohydrates (41.95%) and C fraction of proteins (23.47%) in CH (Table 5). According to Ref.^16^, digestibility may be associated with several factors such as chemical composition, food preparation, kinetics and passage rate in the gastrointestinal tract of animals, animal-dependent factors, and nutritional status.

**Table 5:** Carbohydrate and protein fractionation, and degradation parameters of DM, CP, NDF from cottonseed hull.

	Cottonseed hull
Carbohydrate fractionation (%TC)	
A + B_1_	23.54
B_2_	34.51
C	41.95
Protein fractionation (%CP)	
A	11.28
B_1_ + B_2_	58.16
B_3_	7.09
C	23.47
DM degradation parameters	
a	6.099
b	52.139
c	0.167
CP degradation parameters	
a	5.481
b	89.612
c	0.024
NDF degradation parameters	
a	3.242
b	64.938
c	0.046

A + B_1_ = Non-fibrous carbohydrate (%TC); B_2_ = potentially digestible neutral detergent fibre (%TC); C = unavailable; A = non-protein nitrogen; B_1_+B_2_ = true protein degradable in the rumen; B_2_ = true protein with intermediate degradation rate in the rumen; B_3_ = true protein with slow degradation rate in the rumen, C = unavailable or cell-wall bound true protein; (a) water-soluble fraction; (b) water-insoluble fraction potentially degradable in the rumen as a function of time; (c) rate of degradation per fermentative action of b.

*TC* total carbohydrate.

The apparent digestibility of CP increased by 0.373 percentage units for every 1% of by-product inclusion. According to Ref.^13^, an increase in the digestibility of this nutritional fraction may be related to the diet being kept for a longer period in the rumen, as can be observed by the high value of fraction (c) of DM from CH (Table 5), which gave the microorganisms a longer action time on the food particles, thus increasing PB apparent digestibility. Similar data were described by Ref.^17^ when evaluating intake and apparent digestibility in sheep fed with grains and by-products from canola.

The apparent digestibility of NDF decreased by 0.69 percentage units for every 1% of CH inclusion, which may be related to decreased DM digestibility, which is associated with the decrease in nitrogen availability due to lower CP intake with the increase of CH in the diet. According to Ref.^18^, diets with low nitrogen availability can decrease the digestibility of fibrous constituents from the cell wall due to nitrogen compound deficiency for the ruminal microorganisms’ development.

There was a decrease of 0.60% in the apparent digestibility of EE for every 1% of CH included in the diets, which may be related to the lower content of this nutritional fraction in the CH compared to corn silage (Table 4). The apparent digestibility of NFC and TC were not influenced by the inclusion of CH in the rams’ diet, which may be associated with the animals’ selectivity since the concentrate and corn silage intake was higher for all treatments.

Nitrogen balance decreased with the inclusion of CH in the diets, due to the decrease in CP intake (Table 1) associated with greater nitrogen loss through faeces (Table 2). Nitrogen in the faeces is the sum of indigestible protein from food, bacterial cell nitrogen which multiples in the large intestine using endogenous urea, and peeling. This information corroborates the data in Table 2, which were verified through the C fraction from the protein of CH which presented, on average, 23.47% indigestible protein that is not absorbed by the small intestine in animals and is lost through faeces.

Blood urea (BU) and glucose had averages of 19.23 and 72.35 mg/dL (Table 2). However, it is worth mentioning that the BU concentrations in this research are below those prescribed as ideal for sheep, from 24.0 to 60.0 mg/dL^19^, which shows that protein intake was lower than expected for the animal category used in this research, due to the animals’ selectivity to the diet. However, mean values for glucose were within the reference range for sheep, 50–80 mg/dL^20^.

When evaluating the ingestive behaviour of animals (Table 3), it was observed that in the night-time period (18.00 h to 05.50 h), there was a reduction in the feeding of animals. This probably occurred due to the last offer of feed in the day having been at 15.30 h, and the act of providing food (between 07.30 and 15.30 h) stimulating the animals to go to the trough, resulting in a higher feed intake close to the time of offer since, in this mentioned period, ruminants have a predisposition to look for feed and it is associated with animal selectivity shortly after the offering, resulting in an increase in feeding by animals in the daytime (06.00 h to 17.50 h). This way, at night-time, the animals had no more food to select and reduced their going to the trough.

Daytime rumination was reduced, whereas there was no effect on night-time rumination on inclusion of CH, but when evaluating the periods of the day it was observed that night-time rumination was superior to that in the daytime. According to Ref.^13^, ruminants prefer to feed in the daytime, while at night-time they prefer to ruminate or be idle. Regardless of the time of day, the time spent idling increased with CH inclusion, probably due to a decrease in DM intake as the amount of by-product increased, providing the animals with more time idling.

DM and NDF intake (min/kg) decreased with CH inclusion (Table 1) and caused the animals to spend more time feeding, decreasing DM and NDF feeding rate (Table 6). This is probably because of animals’ selectivity; they preferred concentrate feed and corn silage to CH (Table 4). Lower acceptability of CH in relation to other ingredients in the experimental diets is probably correlated with the lysine and fibre content (structural carbohydrates) that this by-product has in great proportion compared to corn grain and soybean meal (Table 4).

**Table 6:** Ingredient proportions and chemical composition of the experimental diets containing cottonseed hull levels.

Items	Cottonseed hull (% DM)			
	0	10	20	30
Ingredients (g/kg DM)				
Corn silage	500.0	400.0	300.0	200.0
Cottonseed hull	0.0	100.0	200.0	300.0
Soybean meal	74.1	74.2	73.3	71.4
Ground corn	425.9	425.8	426.7	428.6
Chemical composition (g/kg DM)				
Dry matter (g/kg as fed)	270.00	327.28	383.76	439.44
Crude protein	110.04	109.32	108.19	106.66
Ether extract	40.34	38.40	36.49	34.63
Ash	38.88	37.57	36.20	34.78
Neutral detergent fibre ap^1^	284.53	307.92	331.30	354.69
Acid detergent fibre	183.08	205.31	227.49	249.62
Non-fibrous carbohydrate	530.77	510.62	490.99	471.89
Total carbohydrate	813.64	816.91	820.71	825.05
Total digestible nutrients e^2^	744.62	726.90	709.20	691.54

Mineral mixture provided ad libitum—Guaranteed levels (g/kg): 17,7 calcium, 8 phosphorus, 0,4 sodium, 2 sulphur, 5,5 copper, 80 iodine, 12 manganese, 0,15 selenium, 30 zinc and maximum 8 fluoride.

^1^Corrected for ash and protein.

^2^Estimated.

According to Ref.^13^, the rate of ingestion and rumination of DM can be influenced by the particle size and fibre content of food, given the greater difficulty that animals would have in reducing the size of the particles originating from fibrous materials, increasing the retention time of the food and consequently decreasing feed intake. However, it is important to note that the particle size of the CH by-product supplied to the animals is shorter in length and smaller than the grass fibre usually grazed in extensive production systems, thus not only the fibre size but also the type of carbohydrate and its effectiveness should be considered. Research by Ref.^21^ which evaluated the inclusion of babassu cake (0, 7.5, 15, and 22.5%) in the diet of feedlot sheep, showed no effect on DM and NDF eating rate (g/h), so the authors attributed this result to intake of DM and NDF having no effect, a result that corroborates the explanation of a direct relationship between these variables.

The number of ruminated boluses per day and total chewing time (min/day) decreased, probably due to the reduction in DM intake when CH was added to the experimental diets (Table 1). Reference^22^ obtained the opposite result for this variable, that is, an increase occurred in the number of ruminated boluses and consequently in the ruminating activity, which shows the connection between these variables.

The decrease in the time spent ruminating may be associated with low acceptability by rams of CH. The animals selected more concentrate and corn silage than CH (Table 1), thus, due to the lower NDF content, there was a lower rumination stimulus. However, the increase in the time spent idling can be justified by the decrease in rumination activity, and because the feeding time was not influenced by the diet, thus the animals that received the highest level of CH remained idle for longer.

## Conclusion

The inclusion of CH in the diet of feedlot rams reduces intake, the eating and rumination rate, and nutrient digestibility; therefore, it is not recommended to use this by-product in the diet of high-performance rams. However, the use of up to 5% of this by-product for low-yield animals and for maintaining the BW, in general, is justified.

## Methods

### Ethical considerations

Animal procedures were conducted in accordance with the principles oriented towards care in the use of research animals developed by the Federal University of Mato Grosso, Mato Grosso, Brazil. The protocol and methods were approved by the animal research ethics committee of the Federal University of Mato Grosso (No. 23108.046399/13-4). Information provided in the manuscript complies with the essential recommendations for reporting of the ARRIVE 2.0 guidelines (https://arriveguidelines.org/).

### Location, animals, general procedures, and experimental diets

This study was conducted in the metabolism shed of the experimental area of the Animal Science Faculty at the Federal University of Mato Grosso, Rondonópolis Campus, Mato Grosso State, Brazil (16° 28′ S, 50° 34′ W). A total of 24 mixed-breed rams with an average age of 12 months and an average body weight (BW) of 27.6 ± 2.9 kg were allocated to metabolic cages measuring 4.20 × 2 m, equipped with feeders, salt troughs, and drinking fountains. The experiment was performed in May 2015 and lasted 21 days, with 15 days for adaptation to the diets, environment, and management, and 6 days for sample collection.

Samples of ingredients (Table 4) were pre-dried in a forced-ventilation oven at 55 °C for 72 h and ground in a Wiley knife mill (Wiley mill, Arthur H. Thomas, PA, USA) using 1 mm sieves for subsequent analyses of dry matter (DM; method 934.01^23^), ash (method 924.05^23^), crude protein (CP; method 976.06^23^), ether extract (EE; method 945.16^23^) and acid detergent fibre (ADF; method 973.18^23^) Neutral detergent fibre (NDF) and ADF were determined using the methods of Ref.^24^ and had been previously treated with heat stable alpha-amylase^25^.

The NDF corrections for ash and protein (NDFap) were performed according to Ref.^26^. The total carbohydrates (TC) were calculated according to Ref.^27^ the Eq. (1):

1 $$TC = 100 - \left(\%CP + \%Ash + \%EE\right).$$

The non-fibre carbohydrates (NFC) were calculated in the manner proposed by Ref.^28^. Hall by the Eq. (2):

2 $$NFC = 100 - \left[\%CP + \%NDFap + \%EE + \%ash\right].$$

The estimated total digestible nutrient (TDNe) content was obtained according to Ref.^29^ by the Eq. (3):

3 $$TDN: 88.9 - \left(0.779 \times \%ADF\right).$$

For the CH, protein fractions (A, B_1_ + B_2_, B_3_, and C) were determined according to Ref.^26^; carbohydrate fractions (A + B_1_, B_2_, and C) as proposed by Refs.^28,30^ and estimation of the degradation of DM, CP, and NDF (Table 5) according to Ref.^31^.

Diets were formulated according to Ref.^32^ containing 11% CP, prepared for rams weighing 30 kg and an estimated gain of 0.1 kg/day. The diet contained a forage to concentrate ratio of 50 : 50, composed of corn silage, ground corn, soybean meal, and CH. The experimental treatments (Table 6) consisted of the inclusion of 0, 100, 200 and 300 g/kg of CH in diets (DM basis). Mineral mixture and water were offered to the animals ad libitum.

The diets were provided twice a day at pre-established times (07.30 h and 15.30 h). Dietary intake was measured daily by the difference in weight between the food offered and the refusals, which were adjusted in order to correspond to 10% of the total amount offered.

### Intake, digestibility, nitrogen balance, and blood metabolites

Nutrient intake was estimated based on the difference between the total of each nutrient contained in the feed offered and the amount in the refusals, expressed in g/day. Water intake (H_2_OI) was quantified according to Ref.^33^. Composition of the diet effectively consumed was calculated by the relation between the nutrient intake and intake of dry matter (DMI), multiplied by 100.

The digestibility assay was performed by the direct method, through the total collection of faeces and refusals from each animal, twice daily. For collection of faeces, appropriate canvas bags were attached to the animals.

The faeces and refusals were dried in a forced-air oven at 55 °C for 72 h. Then, the samples were ground in a Wiley knife mill with a sieve size of 1 mm and stored until chemical analysis according to Ref.^23^.

The digestibility coefficients (DCs) of DM, CP, NDF, and EE were calculated through the Eq. (4):

4 $$\text{DC}=\frac{ (\text{kg of the portion ingested }-\text{ kg of the portion excreted})}{\text{kg of the portion ingested}} \times 100.$$

In the last 3 days, the urine from each animal was collected to determine the nitrogen balance. Urine collection was performed using buckets arranged under the cages, each containing 50 mL of 50% hydrochloric acid (1 : 1) with distilled water to avoid losses by volatilization of urinary ammonia (NH_3_)^34^. The daily urine volume of each animal was recorded, and a 10% aliquot was taken, packed in plastic flasks, and frozen. Before performing the analyses, all aliquots corresponding to each animal were homogenized. Nitrogen balance (NB) was calculated by the difference between total nitrogen ingested (N ingested) and total nitrogen excreted in faeces (faecal N) and in urine (urinary N). The total nitrogen in faeces and urine was determined according to Ref.^23^ method 976.06.

For determining urea N in plasma, blood was collected on the last day of data collection 4 h after feeding. It was collected from the jugular vein using Vacutainer tubes (EDTA K3 plastic, Osasco, SP, Brazil) then centrifuged at 3000 rpm for 10 min to obtain the plasma, which was stored in a freezer at − 20 °C. The plasma was thawed at room temperature and analysed by a colorimetric enzymatic method in a biochemical analyser (Thermo Plate TP Analyzer, People’s Republic of China) using commercial kits for urea determination (Urea 500, Labtest, Lagoa Santa, MG, Brazil). Plasma urea N concentration was obtained by the product of plasma urea concentration and 0.466, corresponding to the N content in urea^35^. A digital glucose meter (Accu-Chek^®^, Rio de Janeiro, RJ, Brazil) equipped with test tape (tape reagent) was used to read glucose concentrations. Measurements were performed immediately after collecting animal blood^36^.

### Ingestive behaviour

The animals’ feeding behaviour was assessed in the last 3 days of the experimental period. All animals were visually observed for 24 h, and observations were recorded at 10 min intervals, which included feeding, rumination, and remaining idle. Data on each animal’s behavioural activities were recorded by trained observers, who were positioned to interfere as little as possible with the animals’ behaviour. Night-time was from 18.00 to 05.50 h, and daytime was from 06.00 to 17.50 h. During the night period, the environment was kept under artificial light. The eating and rumination rates based on DM (g DM/h) and NDF (g NDF/h) were calculated as described previously^37^. The time and number of chews for each ruminal bolus per animal were recorded (total chewing time min/day).

### Statistical analysis

The experiment was conducted in a completely randomized design with four treatments and five replicates per treatment. The statistical model is represented by the Eq. (5):

5 $$Yi = \mu + \upalpha \text{i} +\beta \left(Xi-\overline{X }\right)+\updelta\text{ij},$$

where Yi was the observed dependent variable, μ was the overall mean, αi was the effect of the diet, *β* was the regression coefficient or functional relationship with the covariate, Xi the observed value of the covariate applied to the experimental unit, $\overline{{\text{X}}}$ the mean of the covariate and δij was the random error.

The data were subjected to analysis of variance through the PROC GLM command of the SAS statistical package (SAS version 9.1 2003), and the means were subjected to regression analysis through the PROC REG command of the SAS^®^ (9.1) statistical package (SAS University Edition). The initial weight was used in the statistical model as a covariate when significant. Analysis of ingestive behaviour also included a t-test to verify the effect of the period (daytime and night-time). Significance was declared when P ≤ 0.05.

## Data Availability

The datasets used and/or analysed during the current study are available from the corresponding author on reasonable request.
